# Efficacy and Safety of Recombinant Human Thrombopoietin on Sepsis Patients With Thrombocytopenia: A Systematic Review and Meta-Analysis

**DOI:** 10.3389/fphar.2020.00940

**Published:** 2020-06-24

**Authors:** Jin Zhang, Zongqing Lu, Wenyan Xiao, Tianfeng Hua, Yao Zheng, Min Yang

**Affiliations:** ^1^The Second Department of Intensive Care Unit, the Second Affiliated Hospital of Anhui Medical University, Hefei, China; ^2^The Laboratory of Cardiopulmonary Resuscitation and Critical Care Medicine, the Second Affiliated Hospital of Anhui Medical University, Hefei, China

**Keywords:** sepsis, thrombocytopenia, recombinant human thrombopoietin (rhTPO), platelet, sepsis-related thrombocytopenia

## Abstract

**Background:**

The efficacy and safety of the administration of recombinant human thrombopoietin (rhTPO) in sepsis patients with thrombocytopenia were still inconclusive.

**Objectives:**

To investigate whether rhTPO is a benefit for sepsis patients with thrombocytopenia.

**Methods:**

PubMed, Cochrane library, Embase, China National Knowledge Infrastructure, and Wanfang Database were electronically searched to the randomized controlled trials (RCTs) from inception to March 4, 2020. The primary outcome was the level of platelet (PLT) on the 7^th^ day of treatment, and secondary outcomes were 28-d mortality, the level of coagulation indicators, hepatic and renal function indicators, blood transfusion, and length of intensive care unit (ICU) stay.

**Results:**

Ten RCTs involving 681 patients were included. For compared with conventional antibiotic therapy, rhTPO could significantly increase platelet counts (PCs) [standardized mean difference (SMD), 2.61; 95% confidence interval (CI), 1.28–3.94; P < 0.001], decreased 28-d mortality [relative risk (RR), 0.66; 95%CI, 0.46–0.97; P=0.03], transfusion volume of blood products and length of ICU stay. Additionally, for compared with conventional antibiotic therapy combined with intravenous immunoglobulin, the pooled results shown that rhTPO also associated with an improvement of PCs on 7^th^ of treatment (SMD, 0.86; 95%CI, 0.54–1.17; P < 0.001), and a reduced transfusion volume of blood products. However, there were no differences in 28-d mortality and the length of ICU stay.

**Conclusions:**

Current evidence shown that rhTPO could increase PCs on 7^th^ day of treatment and reduce the transfusion volume of blood products in sepsis-related thrombocytopenia during hospitalization. The conclusions are needed to be verified indeed by more multicenter RCTs due to the limitation of the included studies.

## Introduction

Although after decades of diagnosis, care, and treatment have improved, sepsis remains a threat to current public health and places a heavy burden on the global economy. Epidemiological studies suggested that the global incidence of sepsis was about 31.5 million and the mortality rate was 16.8% per year (Fleischmann et al., 2016). Thrombocytopenia is a common complication in sepsis patients (Lee et al., 1993; Yu and Yan, 2015; Thiery-Antier et al., 2016), which is called sepsis-related thrombocytopenia (SRT) with the incidence rate of 35%–59% and mortality rate of 13%–83% (Sharma et al., 2007; Levi and Löwenberg, 2008). However, SRT as a complication closely related to the prognosis of sepsis patients, the mechanism and treatment of which are still controversial.

Many factors may contribute to the pathogenesis of SRT (Bedet et al., 2018). Endotoxemia and cytokines in patients with sepsis may activate platelets (PLT) (Marshall, 2010; Schrottmaier et al., 2016), and increase the interaction of platelets with leukocytes, including platelet adhesion (Seeley et al., 2012). Thrombopoietin (TPO) and interleukin (IL)-6 significantly increased in septic patients which promoted the activation of platelet (Shimizu et al., 2018). Platelet counts (PCs) may be reduced observably due to platelet consumption and activation. Besides, thrombocytopenia may be due to the migration of platelets to the lungs, liver, and bone marrow during sepsis (Vincent et al., 2002; Koyama et al., 2018). And the decreased production of platelets and immune-mediated thrombocytopenia may also contribute to the SRT (Larkin et al., 2016). However, the complex mechanism limited the treatment of SRT.

The treatment of SRT involves treating the infection, platelet transfusion, intravenous immunoglobulin (IVIG), and administration of platelet-elevating drugs (Kuter and Begley, 2002; Naime et al., 2018; Critical Care Medicine Committee of Chinese PLA and Chinese Society of Laboratory Medicine, Chinese Medical Association, 2020). Due to the shortage of resources and the risk of blood transfusion, the clinical application of platelet transfusion was limited (Heyman and Schiffer, 1990; Nieken et al., 1995). As we knew, granulocyte macrophage colony-stimulating factor (GM-CSF), recombinant human IL-6 (rhIL-6), and recombinant human IL-11 (rHuIL-11) were used to promote platelet production. However, due to mild thrombopoiesis activity and clinically unacceptable adverse effects, the use of which were also limited (Nieken et al., 1995). And currently IVIG is not recommended for the treatment of SRT (Critical Care Medicine Committee of Chinese PLA and Chinese Society of Laboratory Medicine, Chinese Medical Association, 2020).

Recombinant human thrombopoietin (rhTPO), similar to endogenous TPO, is a recombinant form of the c-MPL ligand, which has been shown to effectively increase PCs (Vadhan-Raj et al., 2005). And it is widely used in chemotherapy or immune-related thrombocytopenia, with curative effects and less adverse effects (Wang et al., 2012; Kuter, 2015). Wu Q et al. reported that rhTPO would increase the PCs in SRT patients and reduce the platelet transfusion effectively (Wu et al., 2014). However, studies also suggested that blocking TPO may be helpful in reducing organ damage in sepsis patients (Cuccurullo et al., 2016; Critical Care Medicine Committee of Chinese PLA and Chinese Society of Laboratory Medicine, Chinese Medical Association, 2020). Thus, it was still inconclusive whether rhTPO can improve the prognosis of sepsis patients with thrombocytopenia. The objective of this study was to clarify the efficacy and safety of rhTPO on SRT by pooled the published randomized controlled trials (RCTs).

## Materials and Methods

The present systematic review and meta-analysis were reported in accordance with the Preferred Reporting item for Systematic Review and Meta-analysis (PRISMA) statement (Liberati et al., 2009).

### Search Strategy

PubMed, Cochrane Library, Embase, China National Knowledge Infrastructure (CNKI), and Wanfang Database were electronically searched to RCTs about rhTPO for treating sepsis patients with thrombocytopenia from inception to March 4, 2020, regardless of language and region. We used the combination of keywords and terms to retrieve each database. In addition, the reference lists of related literature were manually searched for possible trials. The search strategy for PubMed is shown in **Supplementary Table 1**.

### Selection Criteria

Two authors (JZ and ZL) searched independently, according to predefined inclusion and exclusion criteria. First, duplicate literature deletion, title, and abstract screening for relevance were been done using Endnote software. Then, the full-text was acquired to determine inclusion eligibility. Any disagreement would be resolved through discussion, a third review author (MY) would participate in where necessary.

Published literature were included by meeting the following criteria: 1. population: Adult patients with sepsis, severe sepsis, or septic shock, and combining with thrombocytopenia (PLT < 100×10^9^/L) (Shankar-Hari et al., 2016). 2. intervention: recombinant human thrombopoietin. 3. comparison: conventional antibiotic therapy, or the former combined with IVIG. 4. design: randomized controlled trials.

### Outcomes and Data Extraction

Two authors independently extracted data using a pre-piloted form designed by Excel 2019 software (Microsoft Corporation) and the result confirmed by another author. The collected data include: the first author, publish year, study period, sample size, mean age and sex ratio of each group, and the level of PLT at admission, Acute Physiology, Age, Chronic Health Evaluation II (APACHE II) scores at admission, and outcomes data. If any information above is inadequate, we contacted the original author *via* email to consult related data. We resolved discrepancies through discussion. The predefined primary outcomes were the level of PLT on the 7^th^ day of treatment. The secondary outcomes were the 28-d mortality, the length of activated partial thromboplastin time (APTT) and prothrombin time (PT) on 7^th^ day, the levels of glutamic-pyruvic transaminase (ALT) and creatinine (Cr) on the 7^th^ day, the total transfusion amounts of red blood cells, plasma, and platelet during hospitalization, and the length of intensive care unit (ICU) stay.

### Quality Assessment

The quality of filtered articles was been assessed by two authors respectively. Cochrane Handbook for Systematic Reviews of Interventions (5.1.0) was used to assess the risk of bias for RCTs, which contain seven aspects: random sequence generation, allocation concealment, performance bias, detection bias, attrition bias, reporting bias, and other bias (Higgins et al., 2011). We reviewed each RCT and divided them into the high, low, or unclear risk of bias. Trial with more than one high-risk aspect was considered as a high risk of bias whereas trial with low risk of bias for all aspects was considered to be at low risk of bias, otherwise, it was considered as an unclear risk of bias.

### Quality of Evidence

Two authors assessed the quality of each evidence respectively by using the GRADE system (Grading of Recommendations Assessment, Development, and Evaluation) for risk of bias, inconsistency, indirectness, imprecision, and publication bias (Guyatt et al., 2008). The quality was divided into very low, low, moderate, or high. The results were generated by using the GRADE Profiler.

### Statistical Analysis

For dichotomous data, we calculated the relative risks (RRs) with 95% confidence intervals (CIs) by using Mantel-Haenszel method, regardless of the type of effect models. For continuous data, we calculated the standard mean difference (SMD) and 95%CIs. P values less than 0.05 were considered to be significant. Heterogeneity across trials was examined by using the I^2^ statistical tests as well as P values. Those with P < 0.1 and I² greater than 50% seemed as significant heterogeneity, we used a random-effect model to get an overall summary. However, the fixed-effect model would be performed when the result of the heterogeneity test show that P≧0.1 or I²≦50%. The sensitivity analysis was carried out by the leave-one-out method to explore the sources of heterogeneity and tested the stability of results. Publication bias was detected by the funnel plot qualitatively and also quantitatively assessed by using the test of Egger’s. All statistical analyses were performed using Revman software (version 5.3).

## Results

### Literature Research

**Figure 1:**
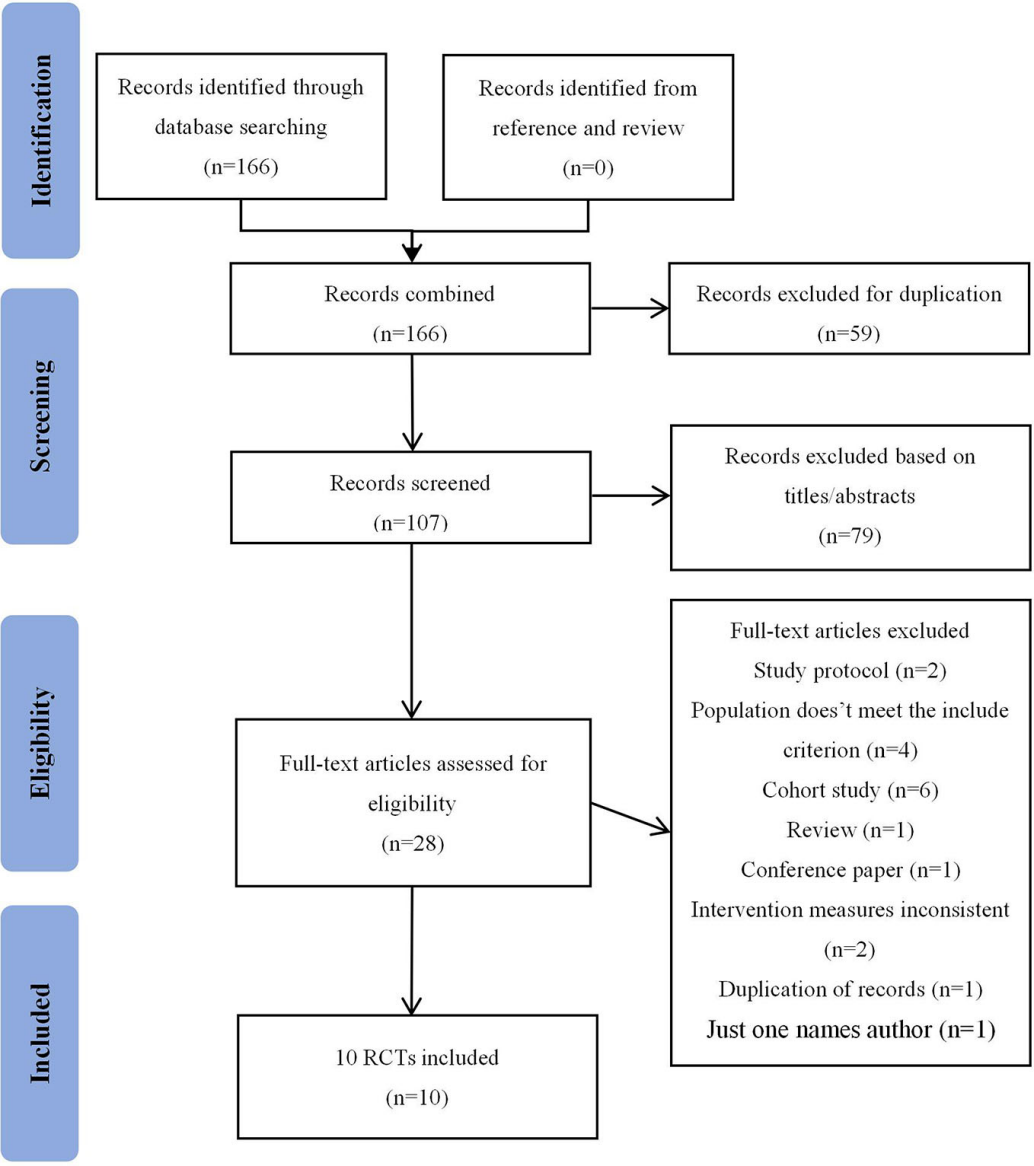
Flow diagram of study selection.

The flow diagram shows the process of literature screening, selection, and reasons for exclusion (**Figure 1**). Our initial search yielded 166 records. After removing duplications and reviewing the titles/abstracts by using Endnote, 28 articles were thought to be potentially eligible for inclusion. After reading the full-text, 18 studies were excluded for the following reasons: study protocol (n=2); population doesn’t meet the criterion (n=4); cohort study (n=6); review (n=1); conference paper (n=1); intervention measures inconsistent(n=2); duplication of records (n=1); only one author (n=1). As a result, 10 studies (Gao et al., 2011; Li et al., 2013; Li, 2015; Yang et al., 2015; Qi et al., 2016; Zhang et al., 2016; Feng et al., 2018; Zhang et al., 2018; Wang et al., 2019; Yan et al., 2019) were eventually included in this meta-analysis.

### Trials Characteristics

Characteristics of included trials were summarized in **Table 1**. The 10 included trials were published from 2011 to 2019, with the sample sizes range from 43 to 102, with a total of 681 participates. For the treatment in control group, 6 trials adopted conventional antibiotic therapy (Li et al., 2013; Yang et al., 2015; Qi et al., 2016; Zhang et al., 2016; Zhang et al., 2018; Yan et al., 2019), 4 trials used the conventional antibiotic therapy combined with IVIG in addition (Gao et al., 2011; Fu and Zhang, 2017; Feng et al., 2018; Wang et al., 2019). The dosage of rhTPO was 300 U/kg/d in most trials, however, there are 2 trials performed 15,000 U/d (Qi et al., 2016; Zhang et al., 2018). There was no statistical difference between baseline data between rhTPO and control groups in each trial.

**Table 1:** The characteristics of included randomized control trials.

First author (published year)	Study period	Sample size		Mean age (Year)		Interventions		The level of PLT at admission (x10^9^/L)		APACHE II at admission		Outcomes
		rhTPO group	Control group	rhTPO group	Control group	rhTPO group	Control group	rhTPO group	Control group	rhTPO group	Control group	
Feng et al. (2018)	2011.09-2013.09	63	39	57.2 ± 21.2	56.9 ± 18.3	300 U/kg/d	CAT+IVIG	28.7 ± 9.7	27.5 ± 14.1	22.6 ± 6.1	23.0 ± 4.6	①③④⑦⑧ ⑨
Gao et al. (2011)	2009.01-2009.11	21	22	43.10 ± 21.25	41.74 ± 17.65	300 U/kg/d	CAT+IVIG	25.14 ± 7.09	26.13 ± 7.11	21.93 ± 8.34	23.47 ± 10.26	①②⑦⑧⑨ ⑩
Li (2015)	2012.01-2014.03	32	35	58.56 ± 25.43	59.09 ± 23.89	300 U/kg/d	CAT+IVIG	36.93 ± 5.50	35.26 ± 4.71	26.94 ± 5.74	24.03 ± 6.35	①②⑦⑧⑨
Li et al. (2013)	2010.01-2011.12	28	20	NA	NA	300 U/kg/d	CAT only	34.78 ± 4.77	36.60 ± 4.25	NA	NA	①②③④⑤ ⑥⑦⑧⑨⑩
Qi et al. (2016)	2015.01-2015.10	30	30	50.3 ± 26.2	50.9 ± 25.7	15,000 U/d	CAT only	52.83 ± 16.32	52.11 ± 16.29	18.8 ± 2.7	18.1 ± 2.2	①②③④⑤ ⑥⑦⑧⑨⑩
Yan et al. (2019)	2016.01-2017.12	42	42	59.13 ± 0.37	59.14 ± 0.39	300 U/kg/d	CAT only	25.49 ± 2.53	25.52 ± 2.51	18.35 ± 2.14	18.31 ± 2.16	①⑦⑧⑨
Yang et al. (2015)	2014.01-2014.12	30	30	NA	NA	300 U/kg/d	CAT only	34.98 ± 0.64	34.31 ± 0.78	NA	NA	①④⑤⑥⑨
Zhang et al. (2018)	2016-2018	34	42	54.50 ± 19.53	53.65 ± 15.52	15,000 U/d	CAT only	30.64 ± 10.19	37.17 ± 1.68	20.21 ± 7.10	19.78 ± 6.05	①②③④⑤ ⑥⑦⑧⑨⑩
Zhang et al. (2016)	2013.10-2015.09	35	31	56 ± 9	54 ± 8	300 U/kg/d	CAT only	37 ± 8	38 ± 19	17 ± 3	17 ± 3	①②⑤⑩
Wang et al. (2019)	2011.09-2013.09	63	39	57.2 ± 21.2	56.9 ± 18.3	300 U/kg/d	CAT+IVIG	28.7 ± 9.7	27.5 ± 14.1	22.6 ± 6.1	23.0 ± 4.6	②⑦⑧⑨⑩

rhTPO, recombinant human thrombopoietin; CAT, conventional antibiotic therapy; IVIG, intravenous immunoglobulin; PLT, platelet; APACHE Ⅱ, Acute Physiology, Age, Chronic Health Evaluation; NA, not available

Outcomes: ① The level of PLT on d7;② 28-d mortality;③ The level of APTT on d7;④ The level of PT on d7;⑤ The level of ALT on d7;⑥ The level of Cr on d7;⑦ The transfusion volume of red blood cells;⑧ The transfusion volume of plasma;⑩ The transfusion volume of platelet;⑩ Length of ICU stay.

### Risk of Bias Assessment and GRADE Profile Evidence

**Supplementary Table 2** shown the details of each risk of bias. On the whole, though, no one in these included trials, had detailed whether blinding for participates, personnel, and outcome assessment was performed, and just two trials reported the allocation concealment. Thus, we had to classify all trials included as unclear risk of bias, according to Cochrane Handbook.

GRADE evidence profiles are shown in **Supplementary Figures 1** and **2**. Overall, the primary outcome was categorized as low-quality evidence. Except for the transfusion of blood products (rhTPO vs conventional antibiotic therapy) were graded as very low-quality evidence due to high heterogeneity, the other secondary outcomes were considered as low-quality.

### rhTPO vs Conventional Antibiotic Therapy

#### Primary Outcomes

**Figure 2:**
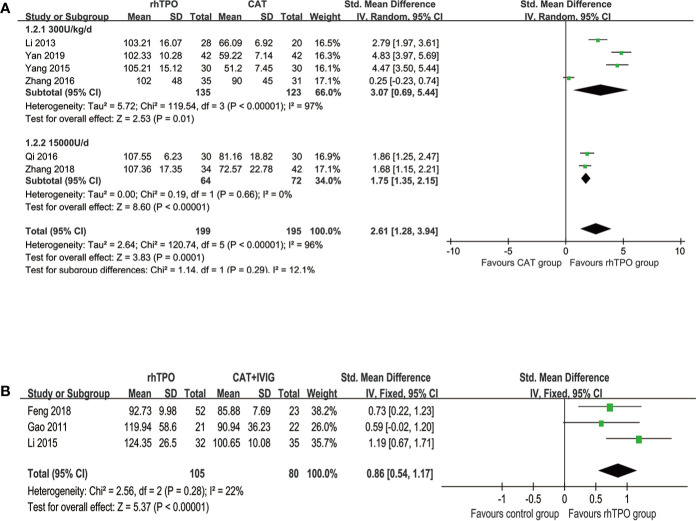
The forest plot for the level of platelet on the 7^th^ day of treatment. **(A)** rhTPO vs conventional antibiotic therapy. **(B)** rhTPO vs conventional antibiotic therapy+ IVIG.

**Figure 3:**
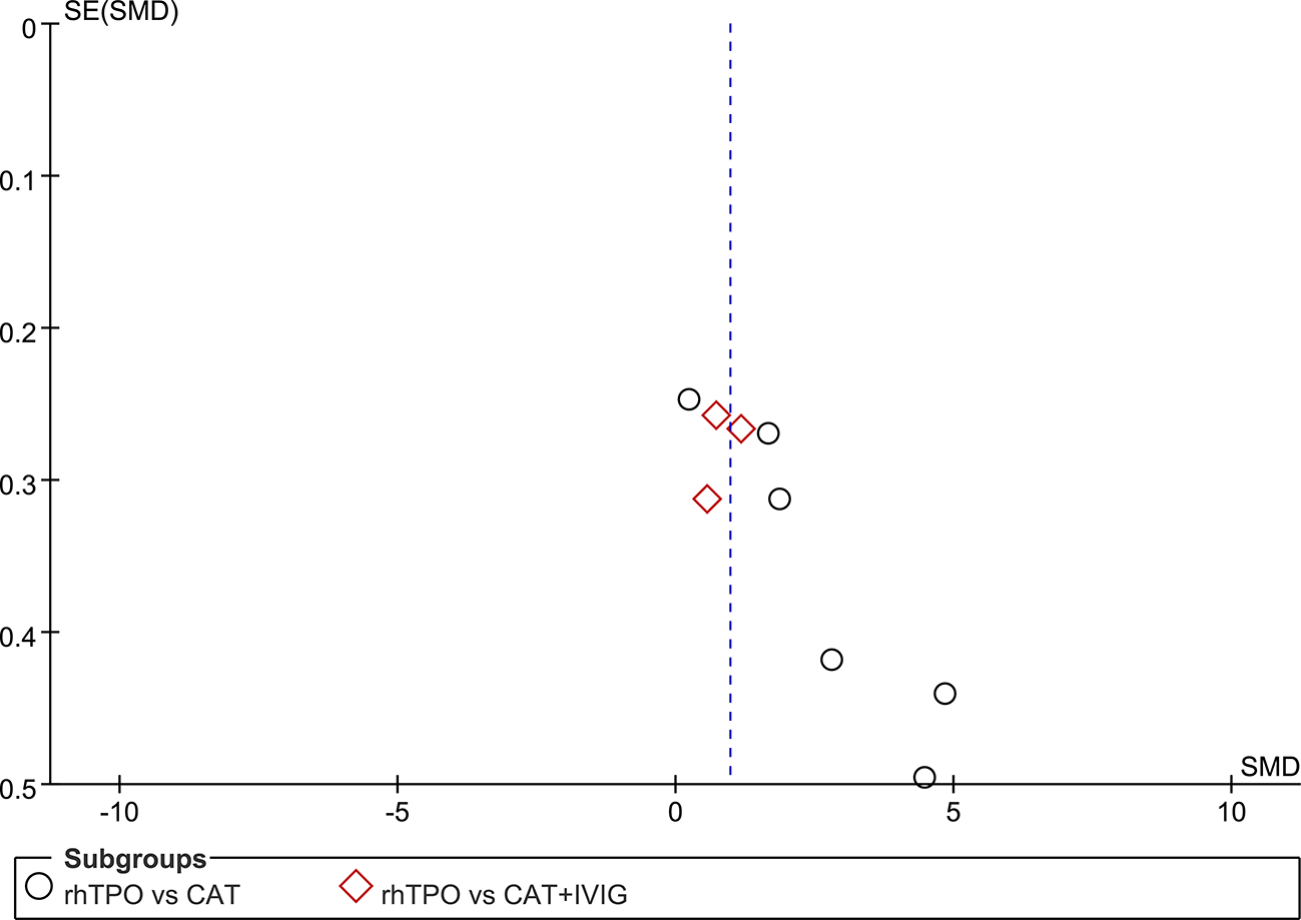
Test for publication bias for the primary outcome.

Six studies reported the information on the level of PLT on 7^th^ day of treatment with totaling 394 patients (Li et al., 2013; Yang et al., 2015; Qi et al., 2016; Zhang et al., 2016; Zhang et al., 2018; Yan et al., 2019). Compared with conventional antibiotic therapy, rhTPO significantly increased the PCs on 7^th^ after treatment (SMD, 2.61; 95%CI, 1.28–3.94; P < 0.001) with high heterogeneity (I^2^ = 96%). Then, we performed subgroup analysis according to different dosages, which found that the results didn’t influence by dosage (**Figure 2A**). However, there was a possible publication bias detected by Egger’s test (P=0.007), the funnel plot was shown in **Figure 3**.

#### Secondary Outcomes

##### The 28-d Mortality

Four trials reported the information about 28-d mortality with totaling 250 patients (Li et al., 2013; Qi et al., 2016; Zhang et al., 2016; Zhang et al., 2018). Compared with conventional antibiotic therapy, rhTPO significantly decreased the 28-d mortality (RR, 0.66; 95%CI, 0.46–0.97; P=0.03) with a low heterogeneity (I^2^ = 40%) (**Supplementary Figure 3**).

##### Coagulation Indicators

Three trials reported the information about the length of APTT (Li et al., 2013; Qi et al., 2016; Zhang et al., 2018) and four trials reported the length of PT (Li et al., 2013; Yang et al., 2015; Qi et al., 2016; Zhang et al., 2018) on the 7^th^ day after treatment. However, there was no significant difference in the length of APTT (SMD, −0.12; 95%CI, −0.41–0.17; P=0.43) and PT (SMD, −0.21; 95%CI, −0.47–0.04; P=0.1) on the 7^th^ day after treatment, when compared rhTPO with conventional antibiotic therapy. And the heterogeneity of these results was very low (I^2^ = 0%) (**Supplementary Figure 4**).

##### Hepatic and Renal Function Indicators

Five trials reported the level of ALT (Li et al., 2013; Yang et al., 2015; Qi et al., 2016; Zhang et al., 2016; Zhang et al., 2018) and four trials reported the level of Cr (Li et al., 2013; Yang et al., 2015; Qi et al., 2016; Zhang et al., 2018) on 7^th^ day of treatment. The results of rhTPO group shown margin effectiveness in the term of ALT reduction (SMD, −0.22; 95%CI, −0.45–0, P=0.05), when compared with conventional antibiotic therapy. However, there was no statistical difference in the level of Cr (SMD, 0.04; 95%CI, −0.21–0.30; P=0.74) between the two groups. The heterogeneity of both outcomes was low (I^2^ = 31% and 0.0% respectively) (**Supplementary Figure 5**).

##### Transfusion of Blood Products

**Figure 4:**
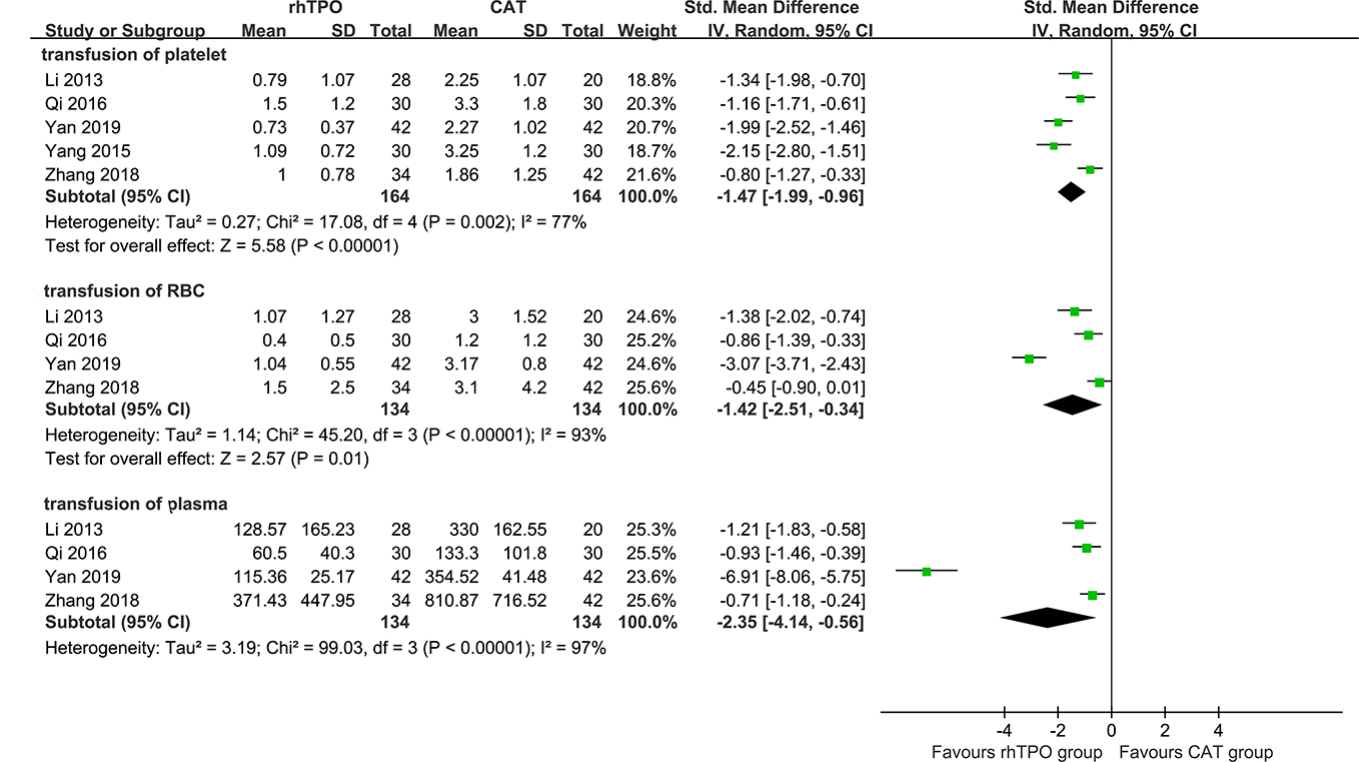
The forest plot for the transfusion of blood products (rhTPO vs conventional antibiotic therapy).

There were five studies reported the transfusion volume of platelet (Li et al., 2013; Yang et al., 2015; Qi et al., 2016; Zhang et al., 2016; Zhang et al., 2018), four studies reported the transfusion volume of red blood cells and plasma (Li et al., 2013; Qi et al., 2016; Zhang et al., 2018; Yan et al., 2019). Comparing with conventional antibiotic therapy, rhTPO significantly decreased the transfusion volume of platelet (SMD, −1.47; 95%CI, −1.99–−0.96; P < 0.001), red blood cells (SMD, −1.42; 95%CI, −2.51–−0.34; P=0.01) and plasma (SMD, −2.35; 95%CI, −4.14–−0.56; P=0.01), and an obvious high heterogeneity was observed in the results (I^2^ = 77%, 93%, 97% respectively) (**Figure 4**).

##### Length of ICU Stay

Four trials reported the length of ICU stay (Li et al., 2013; Qi et al., 2016; Zhang et al., 2016; Zhang et al., 2018) with totaling 250 patients. The pooled result has shown that rhTPO significantly reduce the length of ICU stay (SMD, −0.31; 95%CI, −0.56–−0.0; P=0.02) compared with conventional antibiotic therapy, with a low heterogeneity (I^2^ = 0%) (**Supplementary Figure 6**).

### rhTPO vs Conventional Antibiotic Therapy + IVIG

#### Primary Outcome

Three trials reported the information about the level of PLT on the 7^th^ day of treatment in both rhTPO group and conventional antibiotic therapy combine with IVIG group (Gao et al., 2011; Li, 2015; Feng et al., 2018). The result of meta-analysis shown that rhTPO could increase the PCs on the 7^th^ day of treatment when compared with the control group, and the difference was statistically significant (SMD, 0.86; 95%CI, 0.54–1.17; P < 0.001) (**Figure 2B**), the heterogeneity was low (I^2^ = 22%). No publication bias has been found with Egger’s test (P=0.684) and funnel plot (**Figure 3**).

#### Secondary Outcomes

We just conducted pooled analysis for the 28-d mortality (Gao et al., 2011; Li, 2015; Wang et al., 2019), blood products transfusion (Gao et al., 2011; Li, 2015; Feng et al., 2018; Wang et al., 2019), and the length of ICU stay (Gao et al., 2011; Wang et al., 2019), due to the limited relate data. Comparing with conventional antibiotic therapy combine with IVIG group, the meta-analysis shown that rhTPO could significantly decreased the transfusion of volume of platelet (SMD, −0.65; 95%CI, −0.89–−0.40; P < 0.001), red blood cells (SMD, −0.47; 95%CI, −0.72–−0.23; P < 0.001) and plasma (SMD, −0.61; 95%CI, −0.85–−0.36; P < 0.001), with the low heterogeneity (I^2^ = 0.0%, 49% and 34% respectively). However, the two arms didn’t differ with the respect to the 28-d mortality (RR, 0.82; 95%CI, 0.54–1.24; P=0.34) and the length of ICU stay (SMD, −0.02; 95%CI, −0.35–0.31; P=0.90). The results are shown in **Table 2**.

**Table 2:** The pooled results of secondary outcomes (rhTPO vs conventional antibiotic therapy+IVIG).

Outcomes	Included trials	Heterogeneity		Effects model	Pooled results		
		*P* values	*I^2^*		RR/SMD values	95%CIs	*P* values
28-d morality	3 (Gao et al., 2011; Li, 2015; Wang et al., 2019)	0.7	0.0%	Fixed-effects model	RR=0.82	0.54, 1.24	0.34
Transfusion of platelet	4 (Gao et al., 2011; Li, 2015; Feng et al., 2018; Wang et al., 2019)	0.9	0.0%	Fixed-effects model	SMD=−0.65	−0.89, −0.40	<0.001
Transfusion of RBC	4 (Gao et al., 2011; Li, 2015; Feng et al., 2018; Wang et al., 2019)	0.12	49%	Fixed-effects model	SMD=−0.47	−0.72, −0.23	<0.001
Transfusion of plasma	4 (Gao et al., 2011; Li, 2015; Feng et al., 2018; Wang et al., 2019)	0.21	34%	Fixed-effects model	SMD=−0.61	−0.85, −0.36	<0.001
Length of ICU stay	2 (Gao et al., 2011; Wang et al., 2019)	0.23	31%	Fixed-effects model	SMD=−0.02	−0.35, 0.31	0.90

### Sensitively Analysis

We found that high heterogeneity appeared when rhTPO compared with conventional antibiotic therapy. For the level of PLT on the 7^th^ day of treatment, the I^2^ value decreased to 88% after Yan et al. (2019) and Zhang et al. (2016) excluded, however, the pooled result was stable by using sensitively analysis. For the transfusion of platelet, we found that the I^2^ value decreased to 62.5% when Zhang et al. (2018) excluded. For the transfusion of red blood cells, the I^2^ value decreased to 64% after Yan et al. (2019) excluded, while the pooled result was changed after Li et al. (2013) removed during sensitive analysis. For the transfusion of plasma, we found that the I^2^ value decreased to 0% after Yan et al. (2019) excluded. Thus, we believe that the high heterogeneity may arise from the following factors: sample size, the quality of the included trial, and the difference in dosage of rhTPO.

## Discussion

In this systematic review and meta-analysis of RCTs, compared with conventional antibiotic therapy alone, conventional antibiotic therapy plus rhTPO could significantly increase PCs, and reduce 28-d mortality, transfusion volume of blood products, and the length of ICU stay. And also proved that PCs was improved on the 7^th^ of treatment, reduced transfusion volume of blood products and didn’t increased adverse events when compared with conventional antibiotic therapy combined with IVIG.

The study demonstrated that early control of triggering thrombocytopenia was the prerequisite for treatment (Critical Care Medicine Committee of Chinese PLA and Chinese Society of Laboratory Medicine, Chinese Medical Association, 2020). Even rhTPO would also be a potential therapeutic drug for SRT based on current evidence, effective infection control was the cornerstone of SRT treatment (Larkin et al., 2016). ICU patients with thrombocytopenia are at a high risk of bleeding, receiving transfusions, and death (Williamson et al., 2013). An acute or sustained reduction in PCs always suggests a poor prognosis (Critical Care Medicine Committee of Chinese PLA and Chinese Society of Laboratory Medicine, Chinese Medical Association, 2020). Akca et al. reported that the PCs had been decreased for 14 d, the mortality rate of this disease would be 66% in critically ill patients (Akca et al., 2002). Nijsten et al. also suggested that slow rise of PCs in ICU patients would indicate a worse outcome (Nijsten et al., 2000). In this study, the PCs was significantly improved on the 7^th^ of rhTPO treatment, which may be related to the time required for TPO to promote the proliferation and division of megakaryocytes into PLT (Kaushansky, 2009). Rapid improvement of PCs and shorting of the time to reach the target PLT all would be helpful in reducing bleeding, blood transfusion, and mortality (Akca et al., 2002). Patients with thrombocytopenia always need prolonged vasopressor support and ICU stay (Venkata et al., 2013). The administration of rhTPO could successfully reduce the length of ICU stay and lower the total hospitalization cost due to the effective improvement of SRT (Wang et al., 2019).

Many causes may contribute to the development of thrombocytopenia in ICU. There might be several reasons for rhTPO to increase PCs in SRT patients. First, the production of platelets mainly depends on the maturation and proliferation of bone marrow megakaryocytes, and was influenced by TPO concurrently (Fu and Zhang, 2017). rhTPO can stimulate the formation and differentiation of bone marrow megakaryocytes, and promote the formation of megakaryocytes in all stages, then produces active platelets (Zhang et al., 2016). Studies suggested that rhTPO would promote the proliferation and division of bone marrow megakaryocytes into mature platelets in sepsis, and increased PCs in peripheral blood (Jiang et al., 2019). Second, sepsis involved inflammation initiation and amplification, endothelial dysfunction, platelet activation and aggregation, and coagulation imbalance, which was characterized by the interaction between endothelial cells and activated platelets (Wang et al., 2011; Vardon Bounes et al., 2018). Activated platelets played a key role in the development of sepsis by participating in the activation of inflammation and coagulation pathways (Vandijck et al., 2010). rhTPO might inhibit platelet activation in SRT, weakened the interaction between endothelial cells and activated platelets, and increased PCs (Cloutier et al., 2018). Moreover, PCs may be reduced due to both the platelets’ migration to lungs and liver and bone marrow during sepsis (Vincent et al., 2002; Koyama et al., 2018). Studies have shown that about 14% platelets were sequestrated in the lung tissue in sepsis (Cloutier et al., 2018). The administration of rhTPO could reduce platelet sequestration in sepsis and increase PCs (Jiang et al., 2019). Our results support the application value of rhTPO in SRT patients, and its mechanisms and standardized treatment needs to be further investigated.

IVIG is the main therapeutic drug for immune-related thrombocytopenia (Critical Care Medicine Committee of Chinese PLA and Chinese Society of Laboratory Medicine, Chinese Medical Association, 2020). It is thought to modulate the immune responses associated with sepsis by binding and neutralizing circulating toxins, and also used in SRT (AL-Rawi et al., 2009). However, due the risk of infectious diseases transmission and the high cost of IVIG, the use is limited (Wang et al., 2006). Currently IVIG is not recommended for the treatment of SRT (Critical Care Medicine Committee of Chinese PLA and Chinese Society of Laboratory Medicine, Chinese Medical Association, 2020). In this study, we found that rhTPO was better than IVIG in improving PCs on the 7^th^ of treatment and reducing transfusion volume of blood products, and didn’t increased adverse events. And the cost of the rhTPO treatment is lower than IVIG obviously.

Fever, rash, dizziness, pain at the injection site, and elevated blood pressure were the most common adverse reactions of administration of rhTPO reported in prior studies (Zhou et al., 2015). Furthermore, thrombosis was the main risk of using thrombopoiesis agents (Mahévas et al., 2016). In the all 10 RCTs included, there was no adverse reaction and thromboembolic events reported, which suggested that rhTPO was a safe treatment for SRT.

## Limitations

There were several limitations to this study. First, no blinding was taken place in the studies. But considering the outcome indicators are objective, it may be no impact on results. Second, we found that high heterogeneity appeared when rhTPO compared with conventional antibiotic therapy, and the high heterogeneity may arise from the sample size, the quality of the included trial, and the difference in dosage of rhTPO. Third, the quality of the included literature was low, the sample size was small, and the control was not uniform. In the future, more well-designed RCTs are needed to verify the safety and efficacy of rhTPO on SRT. And the timing of intervention, the course of treatment, the long-term efficacy, and safety need further study. At present, a randomized, multi-center, controlled trial named RESCUE (NCT02707497) is being conducted in Shanghai, which is aims to further investigate that whether the administration of rhTPO is effective and safe therapy on acute severe SRT (Zhou et al., 2019).

## Conclusions

Current evidence has shown rhTPO would increase PCs on the 7^th^ day of treatment and reduced the transfusion volume of blood products in SRT during hospitalization. There was no adverse reaction and thromboembolic events reported in all included studies. The conclusions are needed to be verified indeed by more multicenter RCTs due to the limitation of the included studies.

## Author Contributions

This study was designed by JZ and MY. ZL, JZ, and MY contributed to the literature searching, abstracts reading, data extracting and statistical analyses. The first draft of the essay was written by JZ and ZL. WX and YZ offered some practical suggestions and contributed to the writing of the essay. TH and MY revised the article critically.

## Funding

This study was greatly supported by the funds from the National Natural Science Foundation of China (NO. 81601661) and Natural Science Foundation of Anhui Province of China (No. 1608085MH195).

## Conflict of Interest

The authors declare that the research was conducted in the absence of any commercial or financial relationships that could be construed as a potential conflict of interest.
