# Supplementary figures and images for: Efficacy and Safety of Recombinant Human Thrombopoietin on Sepsis Patients With Thrombocytopenia: A Systematic Review and Meta-Analysis
Source: Front Pharmacol. 2020 Jun 24;11:940. doi: 10.3389/fphar.2020.00940 (PMC7344265; doi:10.3389/fphar.2020.00940)

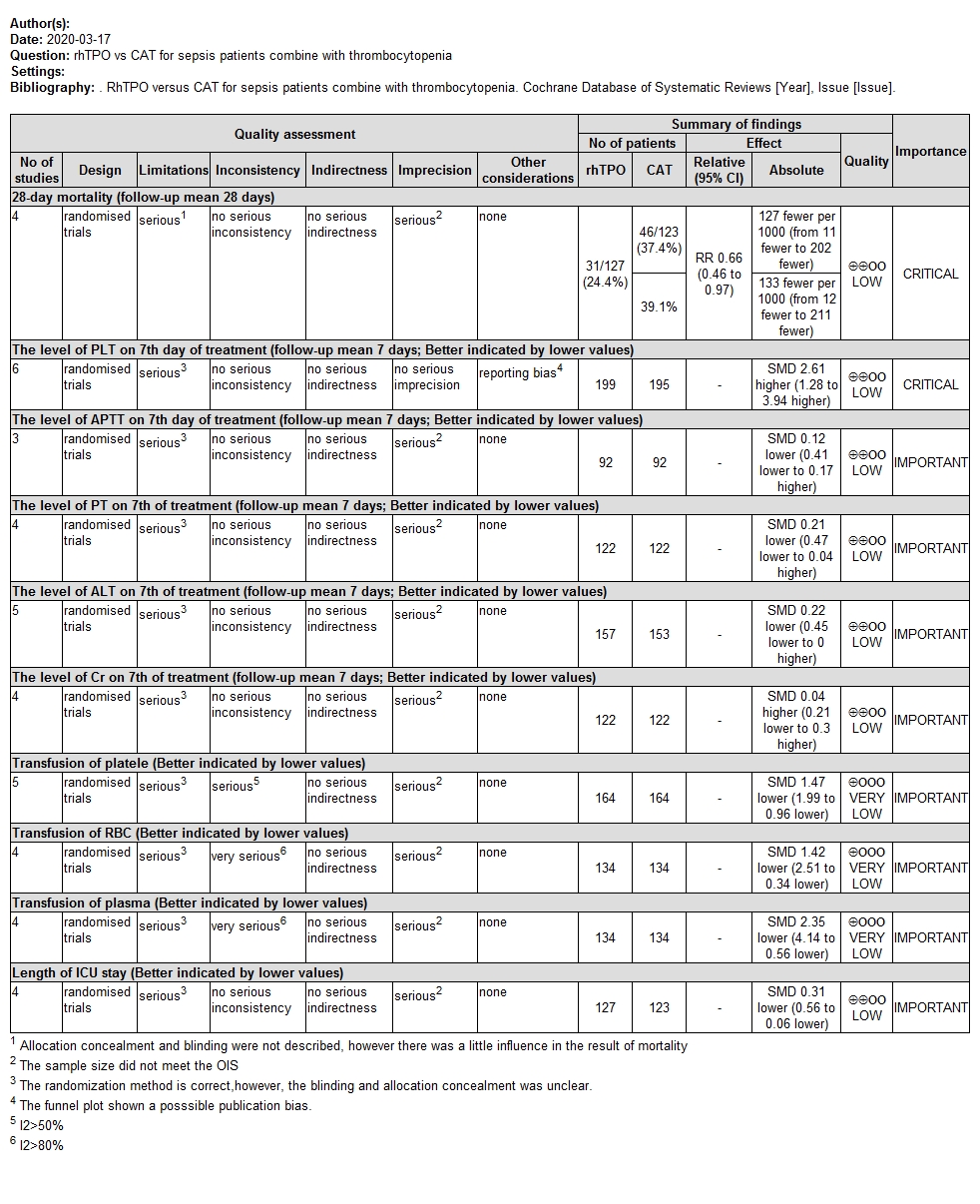

Supplement: Supplementary file 1 [file Image_1.jpeg]

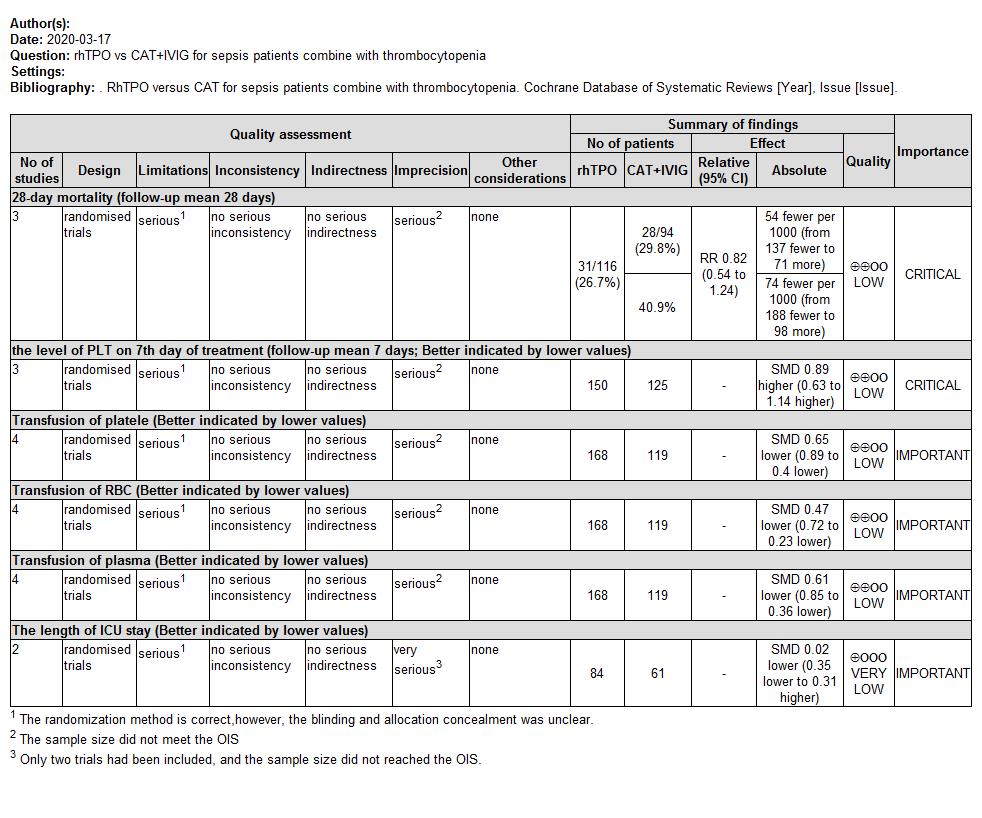

Supplement: Supplementary file 2 [file Image_2.jpeg]

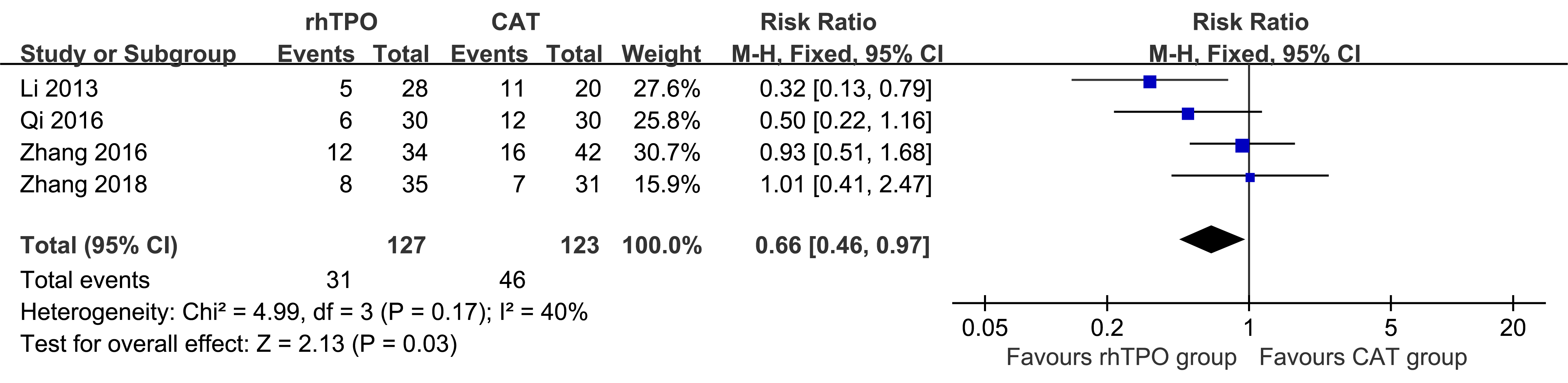

Supplement: Supplementary file 3 [file Image_3.tif]

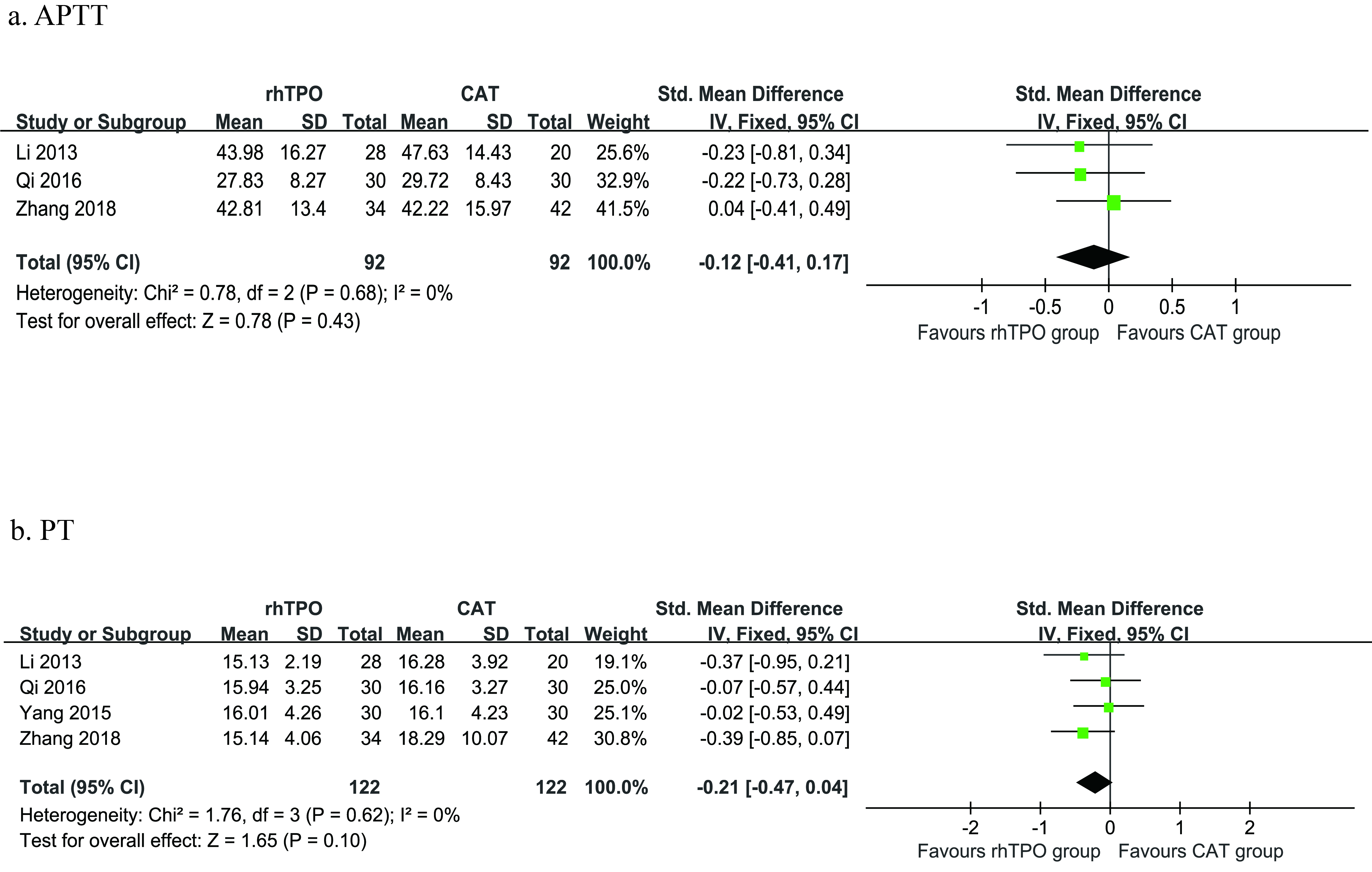

Supplement: Supplementary file 4 [file Image_4.tif]

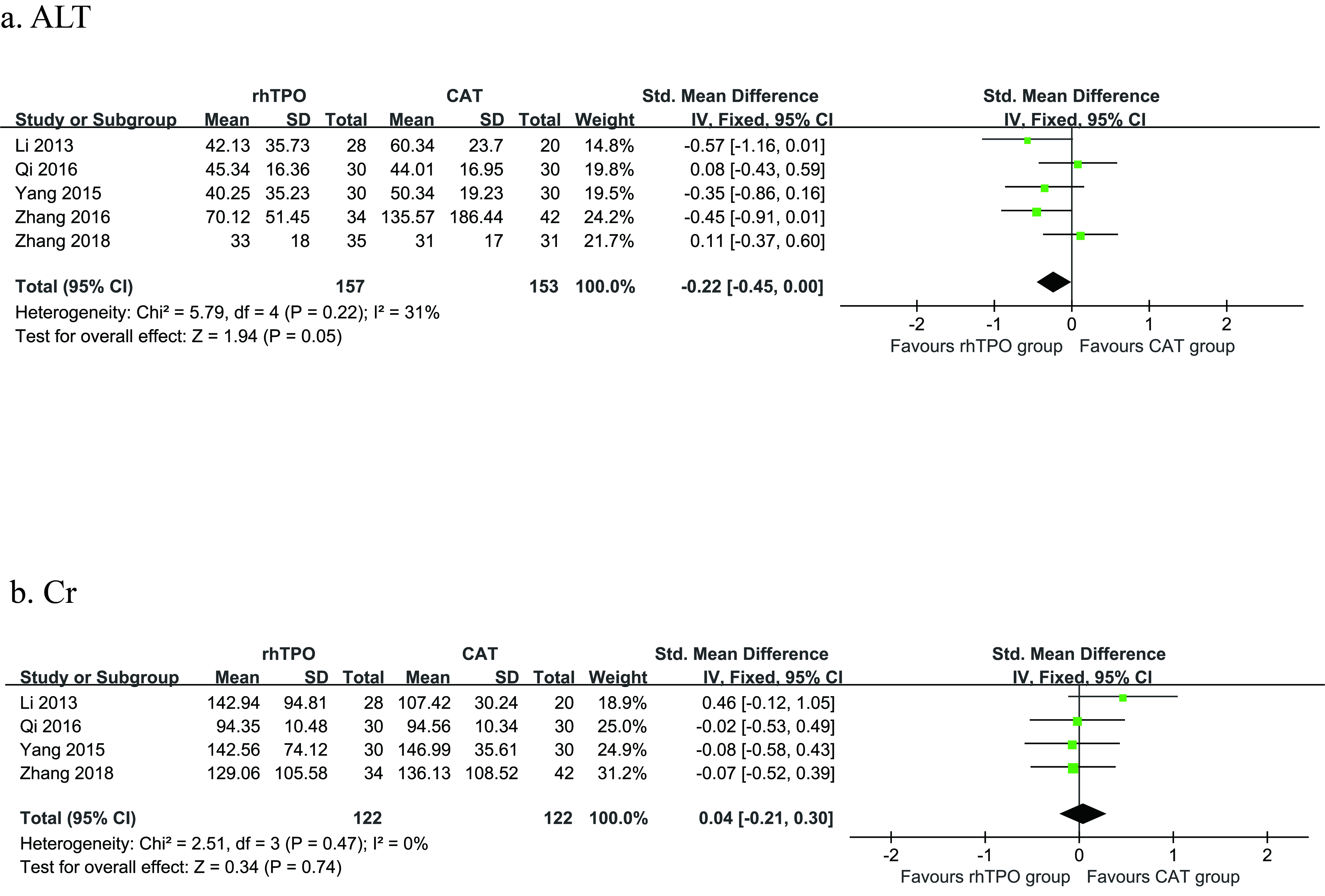

Supplement: Supplementary file 5 [file Image_5.tif]

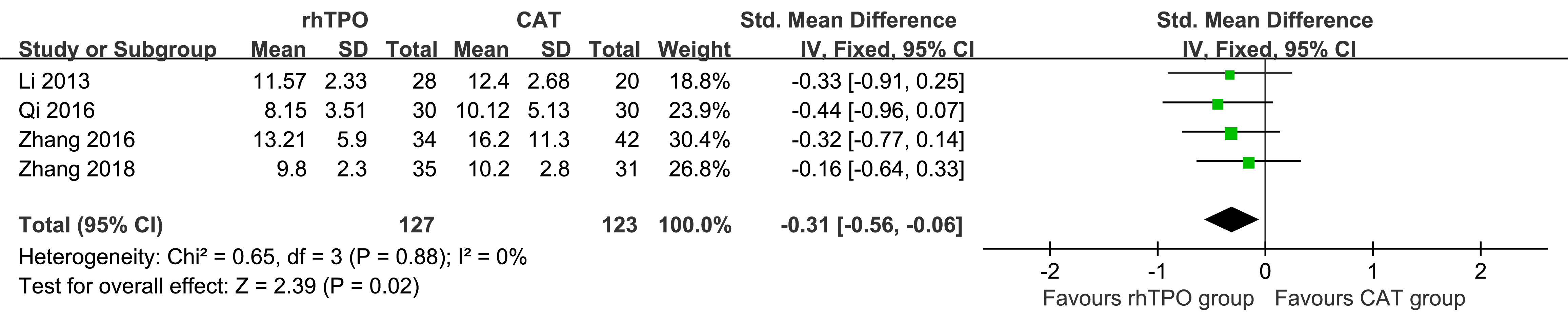

Supplement: Supplementary file 6 [file Image_6.tif]

Supplementary Table 1: The search strategy for Pubmed


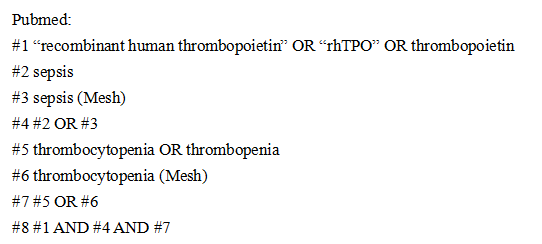

Supplement: Supplementary file 7 [file Table_1.doc]
