# Supplementary material for: Efficacy and Safety of Recombinant Human Thrombopoietin on Sepsis Patients With Thrombocytopenia: A Systematic Review and Meta-Analysis
Source: Front Pharmacol. 2020 Jun 24;11:940. doi: 10.3389/fphar.2020.00940 (PMC7344265; doi:10.3389/fphar.2020.00940)
Supplement: Supplementary file 8 [file Table_2.doc]

Supplementary Table 2: The risk of bias assessment of included randomized control trials.

| First author  (publish year) | Random sequence generation | Allocation concealment | Blinding of participants and personnel | Blinding of outcome assessment | Incomplete outcome data | Selective reporting | Other bias | Overall Risk of bias |
| --- | --- | --- | --- | --- | --- | --- | --- | --- |
| Song-qiao Feng  (2018) | YES | UNCLEAR | UNCLEAR | UNCLEAR | NO | NO | NO | UNCLEAR |
| Hong-mei Gao  (2011) | YES | UNCLEAR | UNCLEAR | UNCLEAR | NO | NO | NO | UNCLEAR |
| Yan Li  (2015) | YES | UNCLEAR | UNCLEAR | UNCLEAR | NO | NO | NO | UNCLEAR |
| Yan Li  (2013) | YES | UNCLEAR | UNCLEAR | UNCLEAR | NO | NO | NO | UNCLEAR |
| Hong Qi  (2016) | YES | UNCLEAR | UNCLEAR | UNCLEAR | NO | NO | NO | UNCLEAR |
| Xi-jiao Yan  (2019) | YES | UNCLEAR | UNCLEAR | UNCLEAR | NO | NO | NO | UNCLEAR |
| Hui-wen Yang  (2015) | YES | UNCLEAR | UNCLEAR | UNCLEAR | NO | NO | NO | UNCLEAR |
| Xiao-ya Zhang  (2018) | YES | UNCLEAR | UNCLEAR | UNCLEAR | NO | NO | NO | UNCLEAR |
| Wei-fang Zhang  (2016) | YES | YES | UNCLEAR | UNCLEAR | NO | NO | NO | UNCLEAR |
| Bing Wang  (2019) | YES | YES | UNCLEAR | UNCLEAR | NO | NO | NO | UNCLEAR |
